# Distinguishing between Zika and Spondweni viruses

**DOI:** 10.2471/BLT.16.181503

**Published:** 2016-10-01

**Authors:** Andrew D Haddow, John P Woodall

**Affiliations:** a United States Army Medical Research Institute of Infectious Diseases, Entomology Department, Fort Detrick, Maryland, 21702-5011, United States of America (USA).; b ProMED-mail, International Society for Infectious Diseases, Brookline, USA.

The Spondweni serogroup includes Zika and Spondweni viruses. Both viruses have been historically misidentified and their diseases have been misdiagnosed due to their serological cross-reactivity and similar clinical presentations. Early case reports indicate a subset of patients present with clinical manifestations suggestive of serious illness.

Flaviviruses have a high serological cross-reactivity. Before the advent of genetic sequencing, serological assays such as virus neutralization and hemaglutination-inhibition were used to differentiate virus species. Much of the early work differentiating flaviviruses into various serogroups was later confirmed by sequencing and phylogenetic analyses. Historically, serological assays (neutralization and complement fixation tests) were also used to determine evidence of prior infection and geographic distribution. Both viruses in the Spondweni serogroup exhibit serological cross-reactivity and cause non-specific febrile illness in humans, making diagnosis challenging in regions where both viruses circulate.

Zika virus was first isolated in Uganda in 1947 (strain MR-766)^1^ and Spondweni virus (strain Chuku) was first isolated in Nigeria in 1952.^2^ Cross-reactivity in neutralization tests led to the misidentification of the Spondweni virus Chuku strain as a strain of Zika virus.^2^^–^^5^ This misidentification led to additional studies where this strain of Spondweni virus was reported as Zika virus, a confusion that continues to the present day, although the misidentification of this isolate was clarified and widely reported in 1964.^3^^–^^5^ Consequently, early clinical case reports from Nigeria,^2^ studies involving the experimental infection of a human volunteer,^6^ vector competence studies in *Aedes aegypti* mosquitoes^6^ and experimental infections in non-human primates,^7^ all involved Spondweni virus (strain Chuku) rather than Zika virus. This cross-reactivity means that the results of early serosurveys — in which only one serological assay was used to screen blood samples from people or animals living in regions where both viruses circulate — are difficult to interpret.^2^^,^^8^

Both Zika and Spondweni viruses are primarily transmitted to humans through the bite of an infective mosquito, and the majority of infections are asymptomatic. In symptomatic Zika and Spondweni virus cases, signs and symptoms appear as early as three days post-infection.^6^^,^^9^ The typical clinical presentation of a Zika virus infection is now well established. The most commonly reported signs and symptoms from 195 patients between 1964 and 2016 are rash (67.2%), fever (63.6%), arthralgia (28.7%), myalgia (23.6%), headache (21.5%), conjunctivitis (20.5%), retro-orbital pain (11.3%), oedema (9.7%), pruritus (7.7%) and fatigue (7.2%).^9^ Less is known regarding the clinical presentation of Spondweni virus infections.

The six well documented cases of Spondweni virus infections report signs and symptoms of fever (100%), headache (83.3%), nausea (83.3%), myalgia (66.6%), arthralgia (50.0%), vertigo (33.3%), conjunctivitis (16.7%), maculopapular and pruritic rash (16.7%), epistaxis (16.7%), photophobia (16.7%), vomiting (16.7%) and disorientation (16.7%).^2^^,^^5^^,^^6^^,^^10^^,^^11^ The only way to distinguish between Zika and Spondweni viruses in regions where both circulate is by confirming a monotypic reaction to a given serologic assay, virus isolation, or detection of viral nucleic acids by polymerase chain reaction.

While most symptomatic Zika and Spondweni virus infections present with mild to moderate febrile illness, a subset of cases present with short duration clinical manifestations suggestive of more serious illness. Before 2014, conjunctivitis, maculopapular and/or pruritic rash, hematuria, hematospermia, aphthous ulcer and epistaxis — indicating vascular leakage — as well as reports of photophobia, vomiting, vertigo, disorientation, meningismus and bilateral transient ocular paresis — indicating neurological involvement — had been reported.^2^^,^^5^^,^^6^^,^^9^^–^^11^ Additionally, Guillain-Barré syndrome, evidence of sexual transmission and evidence of perinatal transmission were already associated with a subset of Zika virus infections.^9^

While Zika virus has a wide geographic distribution,^9^ Spondweni virus has only been reported from sub-Saharan Africa.^12^ Before 2007, both viruses were probably circulating at low levels in sylvatic cycles, causing periodic infections of immunologically-naïve people and non-human primates. It is not surprising that early reports of Zika or Spondweni virus infections are few and they fail to correctly identify which virus is the causative agent. These infections lack clinically distinguishing features; both can present with similar signs and symptoms and most cases occurred in regions with limited infectious disease surveillance, diagnosis and reporting. It is therefore possible that the lack of historic reports of congenital birth defects caused by a Zika virus infection *in utero* may be a result of undetected endemicity: girls exposed before puberty would be immune during their reproductive years.

As infectious disease surveillance activities shift to focus on the current Zika virus epidemic, it is critical to remember that numerous other mosquito-transmitted viruses can cause infections resulting in non-specific febrile illness. These infections may clinically resemble symptomatic Zika or Spondweni virus infections and require appropriate laboratory diagnostics to identify.
